# The Global Pattern of Urbanization and Economic Growth: Evidence from the Last Three Decades

**DOI:** 10.1371/journal.pone.0103799

**Published:** 2014-08-06

**Authors:** Mingxing Chen, Hua Zhang, Weidong Liu, Wenzhong Zhang

**Affiliations:** 1 Key Laboratory of Regional Sustainable Development Modeling, Institute of Geographical Sciences and Natural Resources Research, CAS, Beijing, China; 2 School of Geography, Beijing Normal University, Beijing, China; Universidad Veracruzana, Mexico

## Abstract

The relationship between urbanization and economic growth has been perplexing. In this paper, we identify the pattern of global change and the correlation of urbanization and economic growth, using cross-sectional, panel estimation and geographic information systems (GIS) methods. The analysis has been carried out on a global geographical scale, while the timescale of the study spans the last 30 years. The data shows that urbanization levels have changed substantially during these three decades. Empirical findings from cross-sectional data and panel data support the general notion of close links between urbanization levels and GDP per capita. However, we also present significant evidence that there is no correlation between urbanization speed and economic growth rate at the global level. Hence, we conclude that a given country cannot obtain the expected economic benefits from accelerated urbanization, especially if it takes the form of government-led urbanization. In addition, only when all facets are taken into consideration can we fully assess the urbanization process.

## Introduction

If the transformation of human society since the Industrial Revolution were to be summarized in no more than three words, there would be few better alternatives than industrialization, urbanization, and globalization. These three dimensions have close relations with each other. Industrialization leads to the direct output of economic growth, which further gives impetus to a vigorous process of urbanization in both developed countries and newly industrialized ones, mainly via a specialization of labor and the unprecedented development of non-agricultural sectors. Undoubtedly, the historical facts and statistics reveal that almost all of the developed countries have a higher level of GDP per capita and also a higher level of urbanization. Numerous studies have previously found that the level of urbanization is closely correlated with the level of GDP per capita [1], [2]. It is generally accepted that economic growth promotes the expansion of modern industries and an increase in the urban population; in turn, urbanization also promotes economic growth to some extent. Various programs of accelerated urbanization and rapid economic growth have, therefore, been embarked upon in many developing countries. Policies pursuing positive urbanization, with the goal of boosting economic growth, are widely found in the developing world [3]–[5]. World urbanization is changing quickly and the rate of change has been rising faster in the last three decades than previously, in this age of globalization. Just a few years ago, scholars were saying that more than half of the world’s population would be living in urban areas [6]. Today we hear that the world has entered an urban age, and an urbanization level of 50% has already been reached by the most rapidly developing country, China [7], [8]. The focus of world urbanization has shifted from the developed countries to the developing world.

Much of the literature on the urbanization process and the pronouncements of policy-makers have both hailed growing urbanization as a sign of progress [9], [10]. However, the essence of this interaction is something quite different and more complex. Our understanding of cities is being transformed and, via the new disciplines of complexity science and self-organization theory [11], [12], we now see them as biological systems rather than as mechanical systems. Cities have a strong sense of order and pattern, and are no longer regarded as being disordered systems beneath the apparent chaos and diversity of urban spatial form [13], [14]. Urbanization and urban concentration have a positive impact on economic growth while urban primacy has a negative impact [15], [16]. The argument that urbanization promotes economic growth has recently been challenged by a report showing that there is no evidence that urbanization level affects economic growth rate [6]. This research highlights the importance of re-examining the relationship between urbanization and economic growth, and makes us rethink profoundly the popular ideas and practice of accelerated urbanization in developing countries. More recently, Turok and McGranahan have also argued that it is not urbanization or city size *per se* that induces economic growth, but rather infrastructure and institutional settings [17]. Compelling evidence is still currently lacking, however, and needs to be compiled. First, there has been a substantial change in global urbanization levels and economic development over the past 30 years. This provides a natural checkpoint for verifying whether the existing empirical data support the new view. Second, within the ambit of globalization, most countries are deeply integrated within world systems. Hence, it is desirable to examine the changing global pattern as a whole.

Owing to its impact on the majority of the world’s population and the sustainable development of the global economy, the relationship between urbanization and economic growth is of remarkable scientific and societal importance. We propose to re-examine the arguments that support the view that was widespread in the past or that favor the new vision. The present study aims to address the following questions: (1) What have been the major changes in global urbanization and economic growth over the last three decades? (2) Is there significant evidence that urbanization speed influences the rate of economic growth on a global scale? To answer these two questions, an interdisciplinary methodology for identifying the spatio-temporal pattern is applied to explore the effects of the urbanization process on economic growth, in the context of cross-country panel data derived from the World Bank data sets.

## Data and Methods

Urbanization began during the Industrial Revolution, and refers to the increasing number of people that live in urban areas. Urbanization is not only about a simple increase in the number of urban residents, but also involves a series change from rural to urban styles in terms of industry structure, employment, living conditions, and social public services. Economic growth is the increase in the value of goods and services produced by a country or regional economy over time. Two key indicators are selected to measure development level: gross domestic product (GDP) per capita, and level of urbanization. The research data come from the World Bank’s online database (http://data.worldbank.org/); they are large-scale international data and are freely available to the public. The empirical data cover 226 countries and regions of the world, with yearly observations since 1960. PPP GDP is gross domestic product converted to international dollars using purchasing power parity (PPP) rates. An international dollar has the same purchasing power over GDP as the US dollar has in the United States. GDP at purchaser’s prices is the sum of gross value added by all resident producers in the economy, plus any product taxes and minus any subsidies not included in the value of the products. Data were converted to 2005 international dollars by deflating current local currency units with a Chilean GDP deflator of base year 2005. Using this method GDP per capita data can be compared more accuratelly in ways that eliminate the distorting effect of changes in the Consumer Price Index (CPI). Urbanization level is the ratio of urban to total population. Urban population refers to people living in urban areas, as defined by national statistical offices. It is calculated using World Bank population estimates and urban ratios from the United Nations World Urbanization Prospects. In addition, the purpose of this article is to examine the correlation difference between the level and speed of urbanization and economic growth. Thus, the speed of urbanization and the economic growth rate are computed respectively.
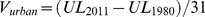


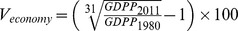
where: *V_urban_* is the average annual speed of urbanization, and *UL_2011_*, *UL_1980_* represent the urbanization levels are for 2011, 1980 respectively; *V_economy_* is the average annual economic growth rate, and *GDPP_2011_*, *GDPP_1980_* represent the GDP per capita in 2011 and 1980, respectively. The index of *V_economy_* is a measure of economic growth from 1980–2011 in percentage terms, providing insight into the general direction and magnitude of growth for the overall economy in each country.

It is widely accepted that GIS and associated analytical software have played a critical role in spatial pattern analysis. The spatial analyst function was used to analyze the global urbanization process and changes in the speed of urbanization and economic growth during 1980–2011. Moreover, to determine whether there is a correlation difference between level and speed in the relationship of urbanization and economic growth, we used the analysis methods of cross-sectional data and panel data, respectively, to test the mutual relationship. If the urbanization level and GDP per capita have a positive relationship, and the speed of urbanization and the economic growth rate are also positive simultaneously. In this case, it provides evidence that urbanization changes of level and speed, no doubt, in the same direction with economic growth. Thus, a positive urbanization policy would undoubtedly be supported. Otherwise, the rationale for pursuing accelerated urbanization would be weakened and would need to be reconsidered. The methodology for the correlation analysis are seen in Appendix S1.

## Global urbanization: patterns of temporal and geographical variation

**Figure 1:**
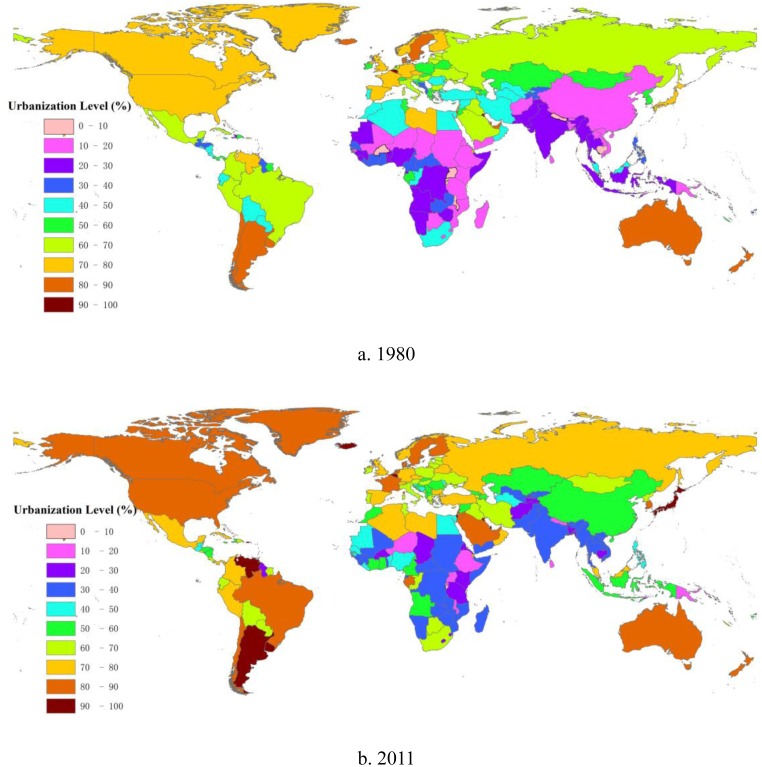
Global patterns of changes in urbanization, 1980–2011. (a) shows the global pattern of urbanization level in 1980, and (b) that observed in 2011. The urbanization level (0–100%) has been divided into ten categories, in blocks of 10%. Each category is denoted by a different color. World urbanization demonstrated remarkable growth in both developed countries and developing countries during 1980–2011, especially in China, Southeast Asia, and Africa.

The attribute data relating to urbanization levels in 1980 and 2011 are put together with the global spatial data and visualized in an GIS environment. Looking at the global distribution of urbanization level, it is easy to see an irreversible trend of world urbanization and remarkable growth in almost all continents during 1980–2011 (Figure 1). Globally, the urbanization level has risen from 39% in 1980 to 52% in 2011. In low- and middle-income groups, the urban proportion has increased rapidly from 31% to 47% over the same period. Interestingly, there is also a growing trend of urbanization in the high-income group, where the level of urbanization has increased from 72% to 80% in 1980–2011. The color difference is clearer in the developing world, especially in Southeast Asia and Africa, which represent the bulk of the urbanization process and where urban population growth has occurred (Figure 1). However, the overall relative level of distribution of urbanization on a national scale worldwide is basically unchanged over the time period. The developed regions, such as North America, Europe and Australia, remain at a higher level of urbanization, while the developing countries are relatively lower. Note that most developing countries in South America have a distinctly higher urbanization level than other developing countries. The urban population ratio of Argentina, for example, reached 92% in 2011, which exceeds the level in the vast majority of developed countries.

We will now interpret the changes in urbanization in more detail, in particular their evolution over the past 30 years, using an approach of classification by different levels of urbanization. Two key indices are employed to compare the differentiation over time: total population and GDP per capita. The urbanization pyramid can provide a useful visual means of analyzing the global pattern, as urbanization level is a key element.

**Figure 2:**
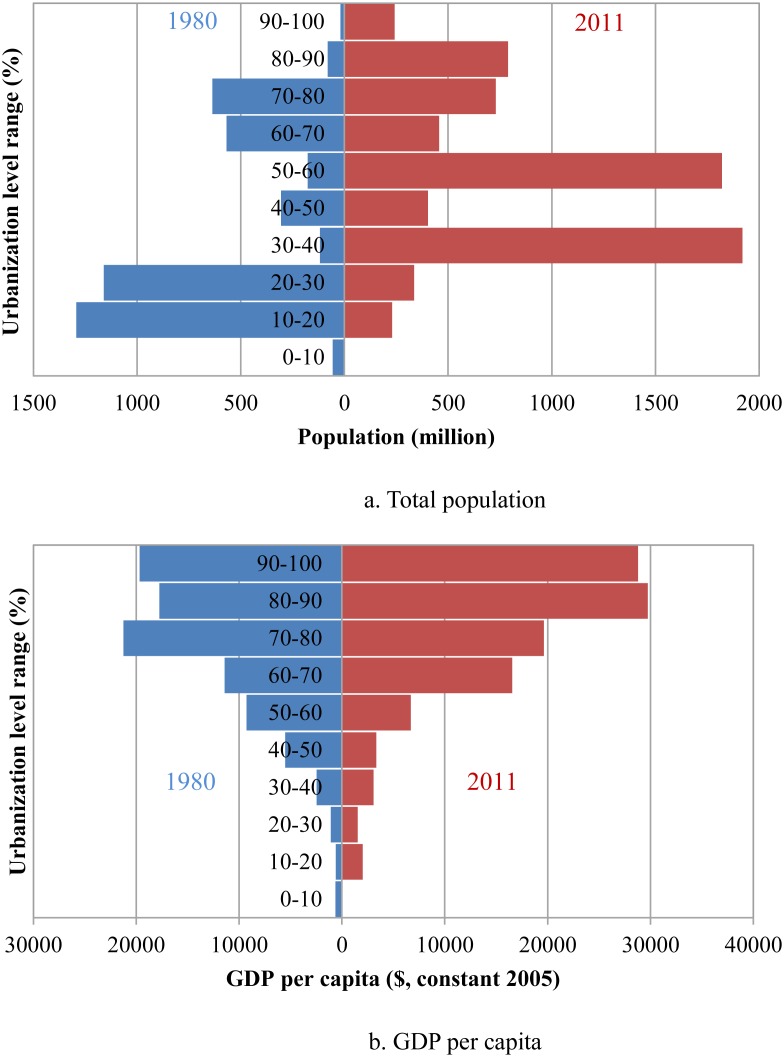
The distributions of total population and GDP per capita by urbanization level. Applying similar population pyramid methods, the structure of total population and GDP per capita are detailed and compared between 1980 and 2011. The blue represents 1980 and the red represents 2011. (a) shows the total population, and (b) shows GDP per capita.

There is significant diversity in the urbanization levels, divided into ten types ranging from 0% to 100%, derived from different countries or regions. In 1980, looking at the global distribution of total population across the urbanization levels, it is easy to see a massive concentration of people between 10% and 30% (Figure 2a), mainly in developing countries, and amounting to 2.45 billion population with a ratio of 55.5% relative to the total population. The total population between 10% and 20% is the highest at 1.29 billion, and includes China, Vietnam, Bangladesh, *et al*. The second highest is in the 20–30% range and has 1.16 billion population, including India, Indonesia, Nigeria, Pakistan, *et al*. The higher level of urbanization is mainly concentrated in the 70–90% range, in developed countries. This band includes the United Kingdom, Canada, the United States, France, Germany, *et al*. The global urbanization level increases remarkably during 1980–2011, and the population peaks are also clear in 2011. The higher peaks of urbanization are in the 70–80% and 80–90% ranges, while the lower levels are in the 30–40% and 50–60% ranges. Thus, the urbanization level extremes of 90–100% also reflected the rising characteristic. Between 1980 and 2011, the world population in the 90–100% range increased by 223 million, growing from 19.2 million to 242.6 million. At the same time, the population in the 0–10% range, which was 57 million in 1980, has changed substantially. No country now falls in the lowest range.

We calculated the value of the average GDP per capita in different groups by considering GDP per capita and the total population of any given country, which provided an accurate description of real development level. Figure 2b shows that urbanization level is closely linked to level of GDP per capita in 1980 and 2011. A higher urbanization level means a higher level of economic development in general, which is similar to what has been reported in previous studies [16], [18], [19]. Moreover, economic growth shows a clear accelerating trend, while the growth in urbanization level increases in each 10% band by between 0% and 70%. In other words, growth of GDP per capita is modest between 0% and 40%, but dramatic between 40% and 70%. It is interesting to note, however, that the average value of GDP per capita is only 3344 dollars in the 40–50% urbanization level group in 2011, even lower than the average value (5507 dollars) in the same group in 1980. There was a similar phenomenon in the 50–60% urbanization level group. We reasoned that, if the urbanization process can drive economic growth, we should observe a higher value of GDP per capita in 2011 in the same urbanization group, at least as large as the original value in 1980. This indicates that the goals of economic growth are often not attained, although some developing countries expect to speed up economic growth via accelerated urbanization, and urbanization level targets are reached. Additionally, the level of GDP per capita in the higher urbanization groups (60–100%) has shown significant growth trends over the last 30 years, while the lower urbanization groups (0–50%) demonstrate a more complicated change in level of GDP per capita. This shows that the gap between countries with higher urbanization levels and countries with lower GDP per capita has been widening during the last three decades.

Finally, there is a very big gap in GDP per capita between the 60–70% and 70–80% groups in 1980, as well as between the 50–60% and 60–70% groups in 2011. Table 1 shows values for the urbanization level, GDP per capita and total population of specific countries in 1980. The level of GDP per capita in the 60–70% group is nearly half that of the 70–80% group, while there is only a 10% difference in urbanization levels between the two groups. The observation that the per capita GDP of Brazil is only 7567, not only far below the per capita GDP of those in the 70–80% group but also much lower than that of the developed countries in the same urbanization level group, calls into question the complex relationship between urbanization and economic growth. The findings also provide evidence that urbanization level is not the key role in economic development.

**Table 1:** Urbanization level and GDP per capita of the 60–70% and 70–80% groups, 1980.

	Country	UL	GDPP	POPU		Country	UL	GDPP	POPU
**60–70%** **urbanization** **level group**	Bulgaria	62.10	5827	8 861 535	**70–80%** **Urbanization** **level group**	Norway	70.55	26 205	4 085 620
	Colombia	62.12	5297	26 874 906		Finland	71.73	17 858	4 779 535
	Dominica	63.41	3921	75 312		Spain	72.79	15 368	37 439 035
	Hungary	64.19	11 347	10 711 122		Germany	72.84	20 861	78 288 576
	Peru	64.57	6083	17 286 832		Bahamas	73.10	26 045	210 600
	Netherlands	64.75	22 271	14 149 800		France	73.28	20 264	55 166 046
	Austria	65.39	20 714	7 549 433		United States	73.74	25 510	227 225 000
	Brazil	65.47	7567	121 711 864		Canada	75.66	23 070	24 593 000
	Saudi Arabia	65.86	33 903	9 801 475		Japan	76.18	17 835	116 782 000
	Mexico	66.34	10 238	68 776 411		United Kingdom	78.48	18 154	56 314 216
	Italy	66.64	18 814	56 433 883		Venezuela	79.19	11 594	15 036 273
	Latvia	67.10	8272	2 511 701					
	Average	64.83	11407	–		Average	74.32	21241	–

The specific countries and developmental indicators are detailed to explain the big gap in the two groups. UL, urbanization level (%); GDPP, GDP per capita (international dollars); POPU, total population. The average GDP per capita is calculated by taking account of the population weight in the same group. It is noted that some countries in Latin America, such as Brazil and Colombia, bring down the averages of GDP per capita in the 60–70% group.

## Global patterns of urbanization speed and economic growth rate, 1980–2011

**Figure 3:**
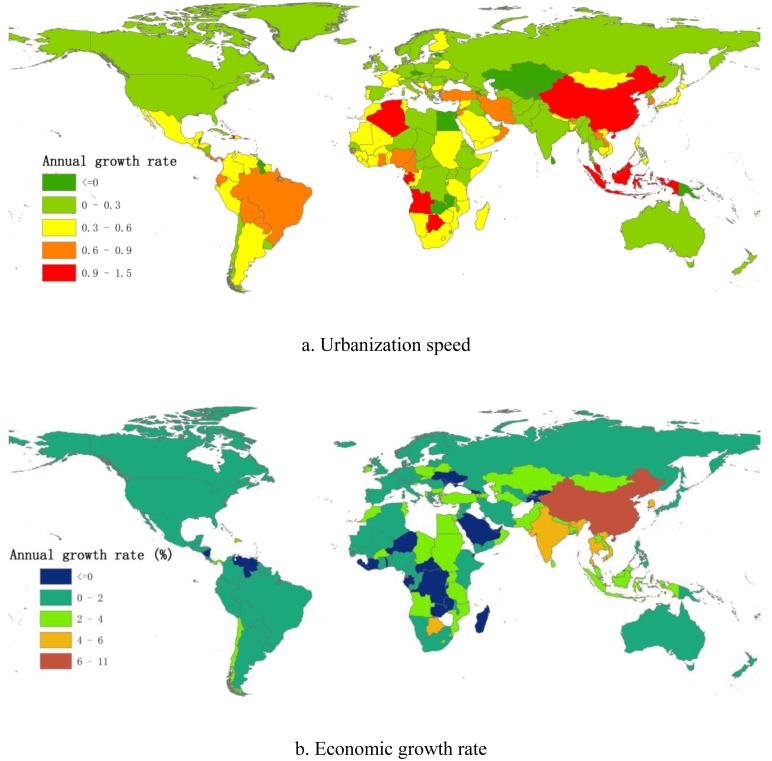
Urbanization speed and economic growth rate, 1980–2011. (a) shows the global pattern of speed of urbanization, and (b) shows the economic growth rate during 1980–2011. Both the speed of urbanization (0–1.5%) and the economic growth rate (0–11%) have been divided into five categories, according to the respective annual increase. Each category is denoted by a different color.

Results are presented in terms of urbanization speed and economic development over the last 30 years. The global pattern of urbanization and economic growth is shown by the average value for the annual growth rate (Figure 3). A statistical analysis was made of groups showing different speeds of development (Table 2). The global patterns details of urbanization speed and economic growth rate are seen in Appendix S2.

**Table 2:** Classification of development speed, 1980–2011.

Urbanization speed					
Classification	Range (%)	Number of regions		Total population in 2011	
		Amount	Ratio (%)	Amount (million)	Ratio (%)
Ultra-high speed	0.9–1.5	13	6.34	1691	24.37
High speed	0.6–0.9	24	11.71	680	9.80
Medium speed	0.3–0.6	65	31.71	1368	19.73
Low speed	0–0.3	75	36.59	2992	43.13
Counter-urbanization	≤0	28	13.66	206	2.97
Total	–	205	100	6937	100
**Economic growth speed**					
**Classification**	**Range (%)**	**Number of regions**		**Total population in 2011**	
		**Amount**	**Ratio (%)**	**Amount (million)**	**Ratio (%)**
Ultra-high speed	6–11	2	1.23	1345	20.12
High speed	4–6	10	6.13	1459	21.83
Medium speed	2–4	48	29.45	1201	17.96
Low speed	0–2	78	47.85	2369	35.44
Negative growth	≤0	25	15.34	311	4.65
Total	–	163	100	6684	100

Detailed classification data are provided for speed of urbanization and speed of economic growth. The number of regions and total population in the different groups also are calculated, clearly demonstrating that low speed is the prevailing trend in both the urbanization process and economic growth.

Over the last three decades, the population with a low annual growth rate (0–0.3%) in speed of urbanization accounted for 44.13% of the global total population. It is interesting to note that counter-urbanization has been observed in some countries, such as Tajikistan, Andorra *et al.*, despite this type only having the lowest ratio to total population.

Additionally, from a comparison of Figure 3a and Figure 3b, it can clearly be seen that China belongs to the ultra-high-speed group in terms of both urbanization process and economic growth. Over the last 30 years, China has had an uninterrupted economic annual growth rate of 8.9%, and a rapid urbanization annual growth rate of 1%. Considering that it is the world’s most populous nation, with 1.344 billion people, China’s transformation is a remarkable and significant achievement [20], not only for China itself but also for global economic development and urbanization, which have benefited greatly from the opening-up and reform policies and from institutional innovations.

## The correlation of urbanization and economic growth

In both the scientific analysis and the development practice of developing countries, the correlation of urbanization and economic growth has been a puzzle to many scientists and policy-makers. Some hold that rapid urbanization always brings economic growth. Others, however, have the distinctly different perception that the two are not necessarily linked. Utilizing the rich empirical data of the last three decades, we will re-examine this puzzle in more detail by distinguishing speed from level and by analyzing cross-sectional data and panel data, respectively.

### Analysis of cross-sectional data

**Figure 4:**
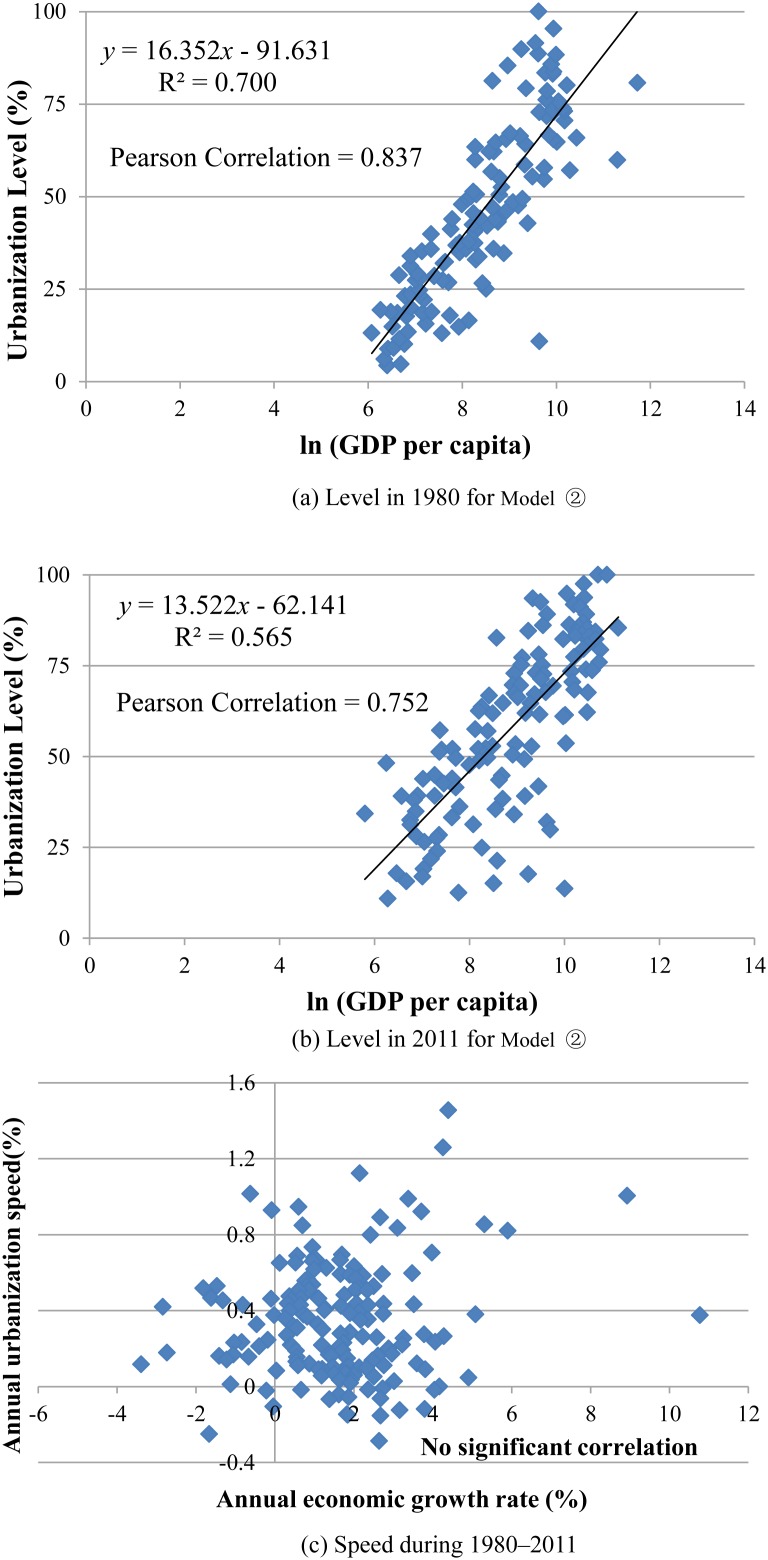
Scatter plots of level and speed of urbanization and economic growth. This figure corresponds to the scatter plot of model 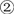, for level in 1980 and 2011, and for speed during 1980–2011. (a) and (b) show the correlation between urbanization level and GDP per capita in 1980 and 2011, respectively. (c) shows the correlation between speed of urbanization and economic growth rate during 1980–2011. Please note the marked correlation difference between level and speed of urbanization and economic growth in the world.

First, we investigate the correlation across countries and regions using cross-sectional level data from 1980 and 2011. The data for average speed are calculated from the level data during 1980–2011. We carry out a regression analysis between urbanization and GDP per capita. While the data are hypothetical, three types of correlation are adopted to represent 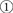 simple linear regression, 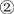 single logarithmic regression, and 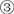 double logarithmic regression. This gives us the basic regression models:
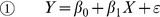
(1)

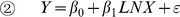
(2)

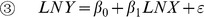
(3)where Y is urbanization level or urbanization speed; X, the level of GDP per capita or the growth rate of GDP per capita. The regression results are reported in Table 3 and Figure 4. The results show that three models comparing urbanization level and the level of GDP per capita for 1980 and 2011 are statistically significant based on the *p*-values of *F*-statistics. From the level perspective, global urbanization and economic development have a positive statistical correlation in both 1980 and 2011. By contrast, the single logarithmic regression model generates the higher value of R^2^ over the other two models at each scenario in level analysis. In case , the urbanization level climbs at a coefficient rate of 16.352 and 13.522 by the unit growth of GDP per capita, with adjusted R^2^ of 0.70 and 0.57 in 1980 and 2011, respectively. This indicates that there is a close link between urbanization level and economic development level. In addition, the Pearson’s coefficients are 0.837 and 0.752 in 1980 and 2011, respectively, which also supports the relevance of urbanization level to development level. This view implies that the urbanization process is, in fact, associated with economic growth in the context of the world pattern. But immediate questions are raised as to whether a necessary correlation exists between the speeds of the two growth processes, and whether accelerated urbanization can bring rapid economic growth.

**Table 3:** The regression results explaining differences between level and speed.

	Level in 1980			Level in 2011			Speed during 1980–2011		
	Y 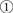	Y 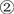	LnY 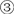	Y 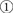	Y 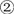	LnY 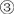	Y 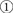	Y 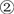	LnY 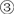
	Model	Model	Model	Model	Model	Model	Model	Model	Model
**X**	0.00086***			0.0011***			0.0219		
	(6.77)			(10.44)			(1.70)		
**LnX**		16.35***	0.436***		13.52***	0.267***		0.00955	0.0213
		(16.93)	(14.87)		(12.64)	(10.41)		(0.30)	(0.22)
**_cons**	37.96***	–91.63***	–0.0172	42.11***	–62.14***	1.579***	0.307***	0.345***	–1.235***
	(17.43)	(–11.17)	(–0.07)	(19.70)	(–6.48)	(6.87)	(9.78)	(11.45)	(–13.77)
***N***	125	125	125	125	125	125	163	138	123
**r2**	0.271	0.700	0.642	0.470	0.565	0.469	0.0176	0.00065	0.000411
**r2_a**	0.265	0.697	0.640	0.466	0.561	0.464	0.0115	–0.0067	–0.00785
**F**	45.79***	286.7***	221.0***	109.1***	159.7***	108.5***	2.878	0.0884	0.0497

*t* statistics in parentheses.

*p<0.05,

**p<0.01,

***p<0.001.

**Figure 5:**
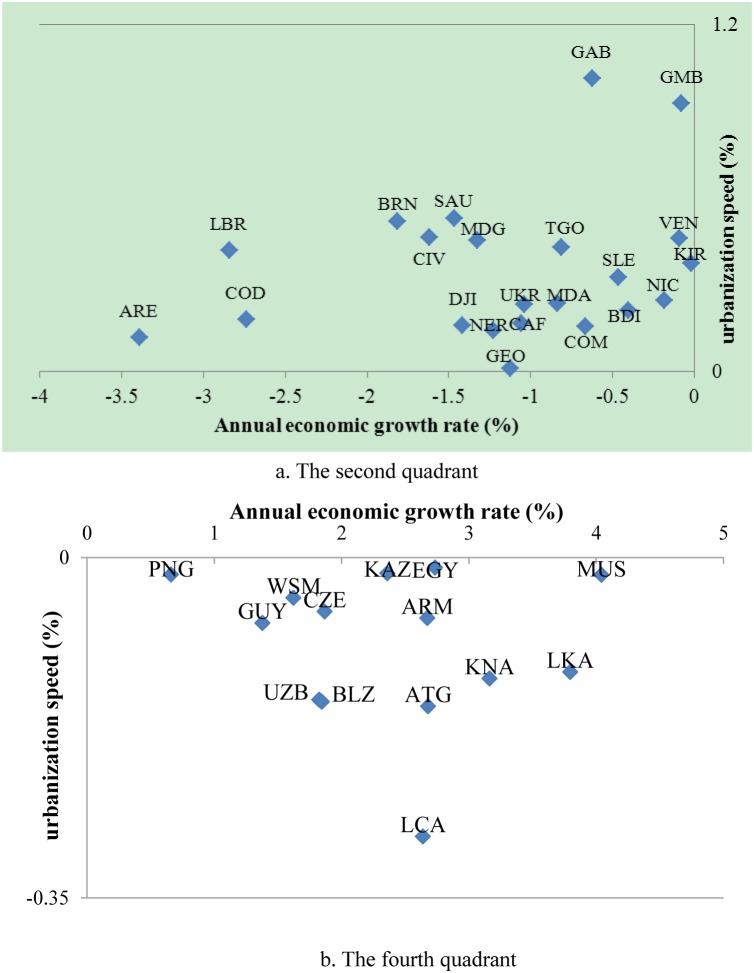
Typical countries demonstrating no significant correlation between speed of urbanization and economic performance. Plotting the annual economic growth rate on the *X*-axis and speed of urbanization on the *Y*-axis, different countries form a set of scatter points on a quadrant map. (a) shows the countries with high urbanization speed and low economic performance, and (b) shows the countries with low urbanization speed and high economic performance. Names of countries are abbreviated to three-digit letters according to the ISO criterion. The full names of the countries are seen in Appendix S3. The results highlight the fact that speed of urbanization has no significant correlation with the economic growth rate of observed common phenomena throughout the world.

In contrast with this close link between levels, however, neither fitting equation is effective in carrying out a regression analysis between urbanization speed and annual growth rate of GDP per capita. Again, using a Pearson’s correlation coefficient test, no significant correlation between urbanization speed and economic growth rate is found (the value of Pearson correlation coefficien is only 0.133, and Sig. (2-tailed) = 0.092). During the past three decades, despite the fact that 22 countries have positive urbanization processes, negative economic growth rates still occur. A vivid case in point is the country of Gabon, which has a high annual urbanization speed of 1.02%, accompanied by an annual economic growth rate of –0.63% in 1980–2011. Meanwhile, there are 14 countries with a negative urbanization speed but a good economic performance. For example, Sri Lanka has realized rapid economic growth at an annual rate of 3.8%, but has undergone a process of counter-urbanization with an annual change rate of –0.12% (Figure 5).

### Analysis of panel data

Because it is efficient to control the influence of heterogeneity of inner unobservable factors, panel regression is more reliable than the cross-sectional data model. To clarify the relationship between urbanization and economic growth still further, GDP per capita and the growth rates of those countries and regions between 1980 and 2011 were introduced into the panel regression.

There are three typical panel models: pooled model, fixed-effects model and random-effects model. The model 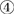 is pooled model, which hypothesizes that if time series and cross-section change, the model intercept and parameter remain constant and not equal to zero. In other words, *α* and *β*_1_ stay constant as *i* and *t* change. The model 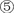 is fixed-effects model, which introduces dummy variables to explain variables. The model 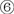 is fixed-effects model. The error term of the random-effects model consists of a cross-section random error component *u_i_*∼N(0, *σ_u_* ^2^), a time series random error component *v_t_*∼N(0, *σ_v_* ^2^) and a pooled random error component *w_it_*∼N(0, *σ_w_* ^2^). For the spurious regression problem of non-stationary time series, stationary and co-integration tests of urbanization rate and GDP per capita were conducted first, and then estimations of pooled model, fixed-effects model and random-effects model were made. The results of the pooled model, the fixed-effects model and the random-effects model are displayed in Table 4 and Table 5, as the respective merits and demerits of the three models were taken into consideration.

**Table 4:** The panel regression results of level data.

	Pooled model (model 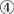 )			Fixed-effects model (model 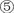 )			Random-effects model (model 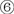 )		
	Y 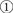	Y 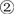	LnY 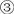	Y 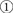	Y 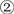	LnY 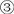	Y 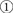	Y 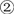	LnY 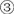
	Model	Model	Model	Model	Model	Model	Model	Model	Model
**X**	0.00118***			0.000346***			0.000370***		
	(54.05)			(17.13)			(18.43)		
**LnX**		14.29***	0.326***		9.183***	0.233***		9.554***	0.241***
		(81.73)	(68.08)		(34.53)	(29.22)		(37.12)	(31.54)
**_cons**	39.00***	–70.65***	1.018***	48.09***	–26.88***	1.816***	47.83***	–30.06***	1.747***
	(105.70)	(–46.60)	(24.53)	(207.50)	(–11.78)	(26.58)	(30.75)	(–11.88)	(23.73)
***N***	3968	3968	3968	3968	3968	3968	3968	3968	3968
**r2**	0.424	0.627	0.539	0.0710	0.237	0.182	0.4566	0.6464	0.5609
**r2_a**	0.424	0.627	0.539	0.0410	0.212	0.155	0.4360	0.6292	0.5425
**F(χ2)**	2921.9***	6679.8***	4634.5**	293.6***	1192.1***	854.0***	339.59***	1378.08***	994.8***

*t* statistics in parentheses;

*p<0.05,

**p<0.01,

***p<0.001.

**Table 5:** The panel regression results of speed data.

	Pooled model			Fixed-effects model			Random-effects model		
	Y 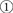	Y 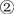	LnY 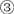	Y 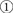	Y 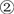	LnY 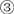	Y 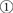	Y 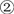	LnY 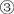
	Model	Model	Model	Model	Model	Model	Model	Model	Model
**X**	–0.000008			0.0000036			0.0000033		
	(–1.03)			(0.61)			(0.55)		
**LnX**		–0.0270***	–0.116***		–0.00241	–0.0131		–0.00447	–0.0212
		(–5.47)	(–9.11)		(–0.45)	(–0.97)		(–0.87)	(–1.61)
**_cons**	0.385***	0.528***	–0.603***	0.383***	0.407***	–1.108***	0.383***	0.412***	–1.145***
	(59.00)	(20.63)	(–9.19)	(88.68)	(15.19)	(–16.37)	(14.00)	(11.22)	(–11.80)
***N***	3844	2775	2466	3844	2775	2466	3844	2775	2466
**r2**	0.000277	0.0107	0.0326	0.0000989	0.0000777	0.000403	0.0043	0.0238	0.0616
**r2_a**	0.0000168	0.0103	0.0322	–0.0332	–0.0467	–0.0525	0.0041	0.0215	0.0602
**F(χ2)**	1.064	29.92***	83.06***	0.368	0.206	0.944	0.3	0.75	2.6

*t* statistics in parentheses;

*p<0.05,

**p<0.01,

***p<0.001.



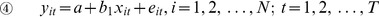
(4)

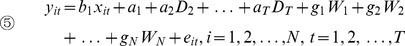
(5)

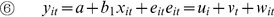
(6)


The panel regression results (Table 4) imply a significant positive relationship between the urbanizing process and the economic process. For the pooled model, fixed-effects model and random-effects model, models 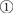, 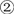, and 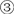 are statistically significant. Because of the larger R2, model 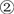 has greater explanatory power, similar to cross-sectional analysis. In all three models, coefficients of GDP per capita are significantly positive, implying the positive relationship between urbanization level and GDP per capita.

The panel regression results for speed data (Table 5) imply that there is no significant relationship between urbanization growth rate and economic growth rate. The fixed-effects model and random-effects model fail in the significance test, while the pooled model passes the significance test, with a goodness of fit of 0.01 and 0.03; this can hardly explain the urbanization growth rate, so there is no significant relationship between the urbanization growth rate and the economic growth rate, which is consistent with cross-sectional analysis.

## Discussion and Conclusions

Careful pattern exploration and regression analysis of global empirical data during the last three decades have allowed a difference to be established between level and speed, and have permitted a re-examination in detail of global variation in the correlation between urbanization and economic growth. Certain countries and regions have already been selected to explore the pattern and relevance of urbanization and economic development [21]–[27]. Our study, however, is the first to conduct a differentiated analysis of levels and speeds of urbanization and economic growth via an approach using a classification of urbanization levels on a global scale in the period 1980–2011. The following key conclusions can be drawn and discussed.

### Changing pattern

The world has experienced an ongoing urbanizing process, and the urbanization level has increased from 39% to 52% in the last three decades as a vast number of people migrate to urban regions. Urban areas play a more important role in national economies worldwide. The urbanization process in developing countries is occurring more dramatically and rapidly compared to that in developed countries. During the study period, the main distributional range has changed from 60–80% to 70–90% in the higher urbanization groups. Meanwhile, the range of lower urbanization groups has increased from 10–30% to 30–40% and 50–60%. The relative patterns, however, do not change the fact that developed nations have a higher level of urbanization than developing regions. Moreover, the ratio of urban population of all the countries in the world was greater than 10% in 2011.

Similar to the Matthew effect, the high urbanization level group often has high levels of GDP per capita, especially in the 0–70% range. The developing countries face a dilemma: after implementing accelerated urbanization to catch up with the developed countries, in 2011 they still had a lower level of GDP per capita than developed countries with the same urbanization level in 1980, in the 40–60% group. Again, the trend of a widening income gap is significant between groups having a high and low urbanization level.

### Correlation analysis

On the one hand, significant correlations of urbanization level with economic development level have been identified in the historic development of any given country, in agreement with the traditional view. On the other hand, no significant correlation has been found between urbanization speed and growth rate of economic indicators in the regression analysis of our rich empirical data set. Though these differential regression results appear, at first glance, to be confusing and contradictory, they exemplify the real and complex association between urbanization and economic development. In the long run, the increasing level of urbanization is a natural consequence of economic development as many rural populations flow to non-agricultural sectors and urban areas. Reasonable urbanization generally also has a positive impact on economic growth [28]. The close link between levels of urbanization and economic growth has resulted in part from the fact that the two processes contain the same evolutional time trend. In the medium to short term, urbanization speed has little effect on economic growth rate. Consequently, accelerated urbanization without parallel economic growth often occurs in the world. It is interesting and noteworthy that a higher speed of urbanization does not, as a rule, lead to more rapid economic growth, as observed in Figure 5. Previously, it has been reported that there is no simple linear relationship between urbanization and economic growth [17]. We argue that the result is robust. While urbanization per se may not lead to economic development, relative or other factors have played an important role in inducing economic growth and raising living standards. For example, urban concentration [2], agglomeration economies [29], and expansion of built-up areas [18] related to urbanization may help to stimulate economic growth and development. Recent cross-country evidence also shows that the potential of urbanization to promote growth is likely to depend on removing barriers to rural–urban mobility, supportive policies, markets and infrastructure investments [17]. To explain the between-country differences, on the other hand, more factors need to be considered, and these may be summarized as geography, history, cultural tradition, governmental management, and institutional setting, which vary between nations. Different countries have aggregated a diverse set of properties. Previous studies have argued that China’s urbanization is unique and bears witness to an essentially endogenous process [4].

### Policy consideration

Our results suggest that a given country will be unable to obtain the expected economic benefits of accelerated urbanization, despite the fact that the country may attain a higher urbanization level by adopting a positive urbanization policy. Indeed, the fact remains that some countries, such as South Korea and China, have achieved rapid urbanization and dramatic economic growth at the same time, creating a global miracle. The explosive growth has stemmed partly from urbanization in terms of economic restructuring and economies of spatial agglomeration. However, there may be other and more immediate reasons that play a more important role in this evolution, such as reform and opening-up policies [30], institutional transition [31] or educational development [32]. Experience has shown that the speed of economic development and urbanization within a given country has little to do with global urbanization speed. Accelerated urbanization is not an automatic panacea for all the ills of economic growth. As for the approach to proper urbanization levels, it is thus inappropriate for decision-makers in developing countries to set too high a target in advance.

Further, urbanization is a complex issue that must be assessed not only in terms of urbanization speed or effects of economic growth. In order to increase the quality of the urbanization process, the forward conditions and backward effects must also be explored. It is suggested that the evaluation of urbanization can be improved from the following two aspects. First, the forward conditions of urbanization can be analyzed, such as the number of non-farm jobs, infrastructure level and the supply capacity of public services. Second, the forward effects of urbanization should be comprehensively evaluated, including economic, social, and environmental sustainability. Numerous studies have shown that urbanization has significant effects on contemporary hot topics, such as CO_2_ emissions [33], [34], climate change [20], water resources [35], biodiversity [36], and human health [37]. Only when all these facets are taken into consideration can we fully assess the urbanization process. Policy-makers in developing countries should seek multiple ways of enabling forms of urbanization that contribute to economic growth, an increase in jobs, environmental sustainability, and so on, rather than pursuing accelerated urbanization.

## Supporting Information

Appendix S1 **The methodology for the correlation analysis.**
(DOCX)

Appendix S2 **Global patterns details of urbanization speed and economic growth rate.**
(DOCX)

Appendix S3 **The full names of the countries using the ISO criterion.**
(DOCX)
