# Supplementary material for: The Global Pattern of Urbanization and Economic Growth: Evidence from the Last Three Decades
Source: PLoS One. 2014 Aug 6;9(8):e103799. doi: 10.1371/journal.pone.0103799 (PMC4123908; doi:10.1371/journal.pone.0103799)
Supplement: Appendix S1 — The methodology for the correlation analysis. (DOCX) [file pone.0103799.s001.docx]

**Appendix S1 The methodology for the correlation analysis**

Correlation analysis is used to verify the relationship between urbanization and economic development by appling several methods(cross-sectional data and panel data models 4-6) and a variety of functional form (model 1-3). Sectional data were first used in the form of three functions (model 1-3). We found that there is a significant correlation between urbanization leveland economic level, while the correlation between urbanization speed andeconomic growth rate is not significant (Table 3).Then, using panel data in all three models (hybrid estimation model, fixedeffects model or random effects model), coefficients of GDP per capita are significantly positive, implying the positive relationship between urbanization level and GDP per capita. Global experience, however, has shown that the speed of economic development and urbanization has little to do with global urbanization speed. The same conclusion is reached by different regression analysis. Thus, we can conclude safely that the empirical findings from cross-sectional dataand panel data support the general notion of close links between urbanization level and GDP per capita. However, we also present significant evidence that there is no correlation between urbanization speed and economic growth rate at the global level.
