# Supplementary material for: The Global Pattern of Urbanization and Economic Growth: Evidence from the Last Three Decades
Source: PLoS One. 2014 Aug 6;9(8):e103799. doi: 10.1371/journal.pone.0103799 (PMC4123908; doi:10.1371/journal.pone.0103799)
Supplement: Appendix S2 — Global patterns details of urbanization speed and economic growth rate. (DOCX) [file pone.0103799.s002.docx]

**Appendix S2 Global patterns details of urbanization speed and economic growth rate**

Speed of urbanization for countries varies significantly, ranging from –0.287% to 1.456% between 1980 and 2011. China, Southeast Asia and some countries in Africa have the highest urbanization speed; South America and some countries in Africa have the highest annual growth rate; and North America, Australasia and some African countries have the lowest growth rate. According to the classification of average urbanization speed values, the countries and regions with ultra-high (0.9–1.5%), high (0.6–0.9%), medium (0.3–0.6%), low (0–0.3%), and counter-urbanization (≤0%) number 13, 24, 65, 75, and 28, and account for 6.34%, 11.71%, 31.71%, 36.59%, and 13.66% of the total, respectively. Using the same standard of classification, the populations of those countries and regions with ultra-high, high, medium, low, and counter-urbanization amount to 1691, 680, 1368, 2992, and 206 million, accounting for 24.37%, 9.8%, 19.73%, 43.13%, and 2.97%, respectively, relative to the total population.

According to the average value of annual economic growth rates, global patterns can be divided into five types as above: 6–11%, 4–6%, 2–4%, 0–2%, and below 0%, denoted as ultra-high speed, high speed, medium speed, low speed and negative growth, respectively. The main characteristics of global economic growth show that the vast majority of countries fall in the range of 0–4% (medium and low speed); they number 126 and account for 77% of the total, and are widely distributed among developed countries and developing regions. The ultra-high-speed category includes only two countries, China and Equatorial Guinea. The high-speed group includes 10 countries and regions, accounting for a population ratio of 21.83%. These are mainly concentrated in Southeast Asia (such as Korea, Thailand and Vietnam), India and some African countries (Botswana and Cape Verde). Some 25 countries, with a population ratio of 4.65%, suffered negative growth in GDP per capita during the period 1980–2011; they are mainly concentrated in Africa.
