# Supplementary material for: The Global Pattern of Urbanization and Economic Growth: Evidence from the Last Three Decades
Source: PLoS One. 2014 Aug 6;9(8):e103799. doi: 10.1371/journal.pone.0103799 (PMC4123908; doi:10.1371/journal.pone.0103799)
Supplement: Appendix S3 — The full names of the countries using the ISO criterion. (DOCX) [file pone.0103799.s003.docx]

**Appendix S3 The full names of the countries using the ISO criterion**

BRN, Brunei Darussalam; BDI, Burundi; CAF, Central African Republic; COM, Comoros; CIV, Cote d'Ivoire; COD, Democratic Republic of the Congo; DJI, Djibouti; GAB, Gabon; GMB, Gambia; GEO, Georgia; KIR, Kiribati; LBR, Liberia; MDG, Madagascar; NIC, Nicaragua; NER, Niger; MDA, Republic of Moldova; SAU, Saudi Arabia; SLE, Sierra Leone; TGO, Togo; UKR, Ukraine; ARE, United Arab Emirates;VEN, Venezuela. ATG, Antigua and Barbuda; ARM, Armenia; BLZ, Belize; CZE, Czech Republic; EGY, Egypt; GUY, Guyana; KAZ, Kazakhstan; MUS, Mauritius; PNG, Papua New Guinea; KNA, Saint Kitts and Nevis; LCA, Saint Lucia; WSM, Samoa; LKA, Sri Lanka; UZB, Uzbekistan.
